# Tailoring dissemination of evidence to preferences of tobacco control partners: results from an academic-community partnership

**DOI:** 10.1186/s13011-022-00450-w

**Published:** 2022-04-22

**Authors:** Virginia Mckay, Mia Vogel, Todd Combs, Laura Brossart, Amy Endrizal, Stephanie Andersen, Timothy Poor, Margaret Mahoney, Douglas Luke

**Affiliations:** 1 Center for Public Health Systems Science, Brown School, Washington University in St. Louis, 1 Brookings Drive, Campus, Box 1196, St. Louis, MO 63130 USA; 2 Health Communication Research Laboratory, Brown School, Washington University in St. Louis, 1 Brookings Drive, Campus, Box 1196, St. Louis, MO 63130 USA; 3 ASPiRE Community Advisory Board Chair, Minneapolis, MN USA

**Keywords:** Dissemination, Policy implementation, Academic-Community partnership, Tobacco control, Health communications, Community advisory board

## Abstract

**Background:**

Tobacco control program leaders and their partners, who often present evidence to policymakers, can increase the use of evidence in program and policy development. However, up-to-date evidence from the scientific community about what works is slow to reach leaders. We describe efforts to understand and utilize tobacco control leaders’ preferences for receiving evidence and report on resulting dissemination strategies, translational products, and outcomes.

**Methods:**

This work is part of the Advancing Science and Practice in the Retail Environment (ASPiRE) Center, an interdisciplinary research center focused on understanding and evaluating tobacco retail policy. Participants were members of the ASPiRE Community Advisory Board (CAB), comprised of tobacco control leaders from 30 metropolitan areas representing all regions of the US plus nine representatives from leading national tobacco control organizations (*N* = 39). During meetings in February 2019 and October 2020, all CAB members were invited to participate in live polls consisting of six survey questions each. Questions addressed preferences for receiving scientific evidence and their anticipated use of ASPiRE translational products. Responses were analyzed descriptively and informed translational product development and communications with ASPiRE contact list members (*N* = 125). ASPiRE email and website interactions were tracked from March 2019 to May 2021 as a complementary indication of content use.

**Results:**

Response rates for 2019 and 2020 CAB meetings were 66% (*n* = 26) and 59% (*n* = 23), respectively. CAB members indicated preferences for email communication (33%) and webinars (31%), communications once per month (46%), and short-format documents (28%). In response, the team developed translational short-format products including case studies, fact sheets, and research briefs. On average, 52% (SD = 14%) of recipients opened the newsletter and 17% (SD = 9%) clicked a link within the newsletter. Overall, 95% of responding CAB members found the products useful and all responding CAB members reported using them to communicate evidence to policymakers, staff, and coalition members.

**Conclusions:**

Our successful dissemination approach to making evidence more accessible and useable for tobacco control leaders could be adapted by researchers working with community partners to assess and respond to stakeholders’ preferences for receiving evidence in other areas of health policy.

## Introduction

Tobacco use has declined significantly in the last two decades, by approximately 60 million people globally [1]. Much of this decline is due to the adoption and implementation of evidence-informed tobacco control policies based on scientific research. The World Health Organization Framework Convention on Tobacco Control, a global treaty open for signature in 2003, and the MPOWER package, a set of evidence-informed strategies to reduce tobacco use developed in 2008, represent early efforts to promote evidence-informed tobacco control polices. As of 2020, 146 countries have adopted at least one MPOWER intervention, protecting 5.3 billion people from the harmful effects of tobacco [2].

Yet even with these notable successes, tobacco use remains a leading cause of preventable death in the U.S. and globally [3, 4]. As of 2019, more than 8 million people die annually from tobacco use and secondhand smoke exposure [5], with more than 80% of people who use tobacco living in low- and middle-income countries [4]. Tobacco control efforts are widely variable across countries, with low- and middle-income countries implementing fewer comprehensive evidence-informed tobacco control policies. In the U.S., tobacco control progress is also unevenly distributed across communities resulting in some communities experiencing higher than average tobacco use and tobacco-related health outcomes [6]. For instance, regional disparities exist in adoption of comprehensive smoke-free policies, with fewer protective policies in rural areas and tobacco-growing states, especially in the South [7]. Many of these same states also lack strong tobacco tax policies [8]. These states have higher prevalence of heart disease, which can be in part attributed to tobacco use.

In the U.S., evidence-informed policies have traditionally proliferated from the ground up, with municipalities serving as test beds for innovative policies [9, 10]. This is especially true in tobacco control policy. For instance, comprehensive smoke-free laws and tobacco retail policies have often been implemented earlier and more widely at the local level before moving to federal levels [11]. As of January 2021, more than 1100 local and 28 state comprehensive smoke-free laws covered 60% of Americans [12].

However, the effective adoption and implementation of evidence-informed health policies is often a complex and painstaking process, even at the local level [13]. Differences in culture between policy makers and scientists, complex scientific outcomes, and the applicability of the evidence to current, often rapidly evolving public health issues are among the factors that make evidence-informed policy more difficult﻿ [14]. Within the U.S., tobacco control leaders (e.g., health department program managers, health commissioners, and advocates) at state and local levels represent potential bridging actors between researchers and policy makers to help integrate scientific evidence into policy development [15, 16]. Tobacco control leaders are responsible for implementing health programs and policies, developing policies for public health issues prioritized by elected officials, and presenting policy impacts to elected officials. As such, targeting tobacco control leaders as part of dissemination efforts by scientists has the potential to enhance the integration of scientific evidence into policy.

Active approaches, which push evidence towards end users, can facilitate the successful integration of evidence into health policy by tobacco control leaders [13, 17, 18]. As an initial step, assessing intended audience preferences can help guide researchers’ efforts to disseminate evidence through channels accessible and preferable for the intended audience (e.g., email, websites, or hard copies) and in a format that is understandable and usable for the intended audience (e.g., briefs or infographics rather than scientific articles). A growing body of research within dissemination and implementation science suggests a number of preferences to consider while designing and tailoring dissemination approaches including:

- The needs and characteristics of the intended audience, for example, limited available capacity and time to digest and synthesize the body of scientific literature on a particular health issue among policy makers [19].
- The presentation of evidence in a format and dissemination channel that is accessible and desirable. Some policy audiences prefer short documents while others prefer full-length scientific articles. Some prefer electronic documents while others prefer paper documents. For instance, in a previous survey of 12 state health departments and affiliated partner organizations, participants preferred receiving research evidence through webinars and workshops and most frequently used research findings for assessment and evaluation, funding, and intervention selection [20].
- The content of the evidence and its relevance to the public health issue. For instance, presentation of a story-like narrative rather than lengthy statistical presentations, data visualizations over tables, and cost-effectiveness evidence. Information that is clear and actionable with recommendations [21]﻿.
- Organizational and community factors like community opinion about a particular public health issue [22].

However, there are few practical and feasible examples of how to use community stakeholder preferences to guide dissemination efforts among tobacco control leaders and tobacco control policy audiences more generally.

We report our efforts to translate and disseminate scientific evidence about tobacco control policy through the Advancing Science and Practice in the Retail Environment (ASPiRE) Center, a National Cancer Institute–funded interdisciplinary tobacco retail policy research center consisting of three major research projects and additional supportive cores. ASPiRE aims to build a strong scientific evidence base for effective retail policies to help reduce tobacco use, tobacco-related disparities, and the public health burden of tobacco use [23]. As part of the ASPiRE Center, the Dissemination and Implementation Core (D&I Core) works to improve the communication, adoption, and impact of ASPiRE-generated research as well as other emerging scientific evidence by generating translational products. The D&I Core's work is informed by Brownson and colleagues’ Designing for Dissemination and Sustainability(D4DS) principles [24, 25]. Building on Designing for Dissemination, The D4DS logic model describes principles to design research studies with dissemination in mind, rather than waiting until a project is nearly finished, so that evidence is disseminated sustainability and equitably for long-term impact. Included in the model are four phases: conceptualization, design, dissemination, and impact. In alignment with the D4DS logic model, the research focus of ASPiRE was conceptualized with tobacco control policy makers in mind and their expressed evidence needs. We report on the subsequent three steps: design, dissemination, and early impact of ASPiRE research. Of particular relevance to the desribed work, the D4DS elevates the design portion of the model to consider the package, or format of the evidence in a product, and distribution in a manner that fits with the needs and preferences of the intended audience [25].

Central to this effort is partnership with a community advisory board (CAB) of tobacco control leaders, directors, and advocates. The ASPiRE Center researchers engage with the CAB formally on a bi-annual basis and informally so the CAB can provide guidance to ASPiRE research teams about retail policy activity and research needs, provide feedback on data collection instruments, identify sources of data concerning licensed tobacco retailers in participating cities and counties, and help disseminate research findings and policy tools to the field. As part of these meetings, the D&I Core team provides updates about new tobacco control science and materials available to CAB members and gathers information from CAB members about evidence needs.

With the goal of enhancing the translational pipeline to increase use of scientific evidence in tobacco control policy and providing a model of how this might be accomplished, we describe our efforts to understand CAB members’ preferences for receiving evidence. We then provide examples of how we used reported preferences to develop translational materials and shape dissemination efforts. Finally, we describe CAB members’ reported engagement and use of disseminated materials.

## Methods

The work for the current study was carried out in three phases. First, we conducted a survey eliciting information from CAB members regarding their preferences for scientific evidence. We then developed a variety of translational products to disseminate scientific evidence in response to the outcomes of CAB members’ reported preferences. We conducted a follow up survey with CAB members to understand whether and how they used the disseminated products. We also tracked usage on two of our developed products the ASPiRE website and a bi-monthly newsletter. This project was determined to be non-human subjects research by the Institutional Review Board at Washington University in St. Louis.

### Participant Eligibility and Recruitment

**Fig. 1:**
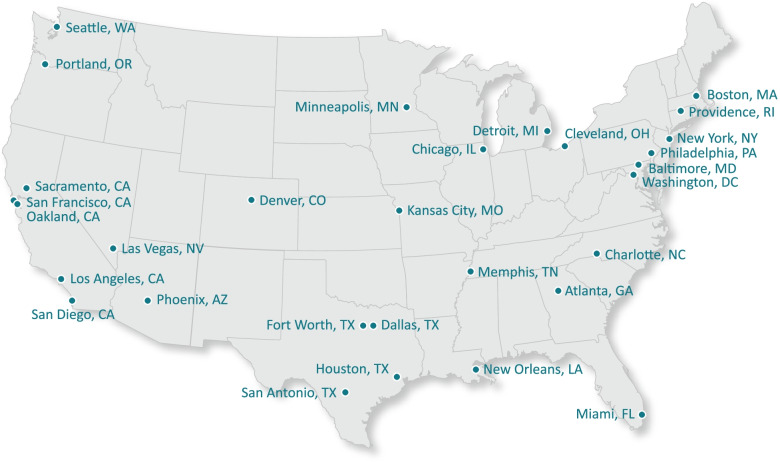
Cities represented on the ASPiRE CAB

Recruited participants were members of the ASPiRE Community Advisory Board (CAB). The CAB is comprised of tobacco control leaders from 30 large metropolitan areas representing all regions of the United States and several representatives from leading national tobacco control organizations (e.g., Campaign for Tobacco-Free Kids, the Centers for Disease Control and Prevention’s Office on Smoking and Health, & the Public Health Law Center) and consists of approximately 45 members in total. Metropolitan areas represented on the CAB are illustrated in Fig. 1. Most of the 30 selected cities are part of the Big Cities Health Coalition [26]. Invitations to participate on the CAB were extended to tobacco control leaders from two southern cities (Atlanta and New Orleans), for geographic diversity, and Providence due to the city's early adoption of novel policies.

We used existing networks and past experience to identify an individual from each city who would be best suited to participate. If an individual was not easily identified, we relied on advice from the state health department or health advocacy organizations (e.g., the American Cancer Society, the American Heart Association, or the American Lung Association). All invited city representatives are engaged in tobacco control policy work. Most serve in local public health departments, with a few employed by the state health department or a non-governmental organization. Most have leadership roles in either chronic disease, community engagement, or tobacco control policy work within their organizations, though some are highly engaged staff-level personnel. In the instance of turnover, some members recommended their own replacements; in other cases, the CAB lead found a replacement who has deep knowledge and concern for tobacco control policy.

## Data Collection

**Fig. 2:**
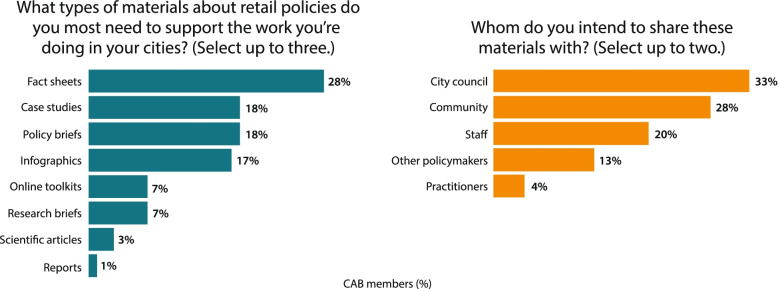
Product type preferences and intended audiences for products, 2019

Data were collected during our first and second CAB meetings in February 2019 and October 2020, respectively. All CAB members in attendance were invited to participate in a live poll using Poll Everywhere online software [27]. At each meeting participants were surveyed using six close-ended, multiple-choice questions. Questions and response options are provided in Figs. 2 and 4. Questions were developed by D&I Core team members in conjunction with the CAB chair. The conclusion of the questionnaire provided an optional write-in section for participants to provide additional thoughts or feedback. Questions asked for the February 2019 meeting focused on dissemination preferences and intended uses of disseminated products. In October 2020, questions focused on actual use of disseminated products. Participation in the questionnaires was completely voluntary, each question was optional, and participation was anonymous. Potentially identifying information (e.g., participant mentioning their location or organization) was removed.

### Data analysis

Results from the questionnaires were managed and analyzed using R statistical programming language version 4.0.3 [28]. At each time point, descriptive statistics were reported for questionnaire response options including absolute frequency, relative frequency, and an error term, where appropriate. Questionnaire results were visualized for ease of interpretation.

## Results

Following a short description of CAB meetings and participants, we report findings from surveying CAB members on their dissemination preferences. We then describe the translational activities and products developed in response to those preferences. We also provide CAB member self-reported use of the evidence and results from an evaluation of CAB member satisfaction with the tailored dissemination efforts.

### CAB Member Participation and Responses

Thirty-three of 39 CAB members attended the February 2019 in-person meeting. Twenty-three of 39 CAB members attended the October 2020 meeting held remotely via Zoom. Response rates for 2019 and 2020 CAB meetings were 66% (*n* = 26) and 59% (*n* = 23), respectively. Most individuals served at the manager or director level in their organizations.

### Dissemination Preferences

Respondents indicated a preference for receiving information primarily by email (33%), followed closely by webinars (31%) over other channels to hear about new retail policy resources. There was also interest in receiving information through websites, blogs, and conferences. Participants indicated very little to no interest in receiving information through social media or mail. Many (46%) preferred an update once per month.

**Fig. 3:**
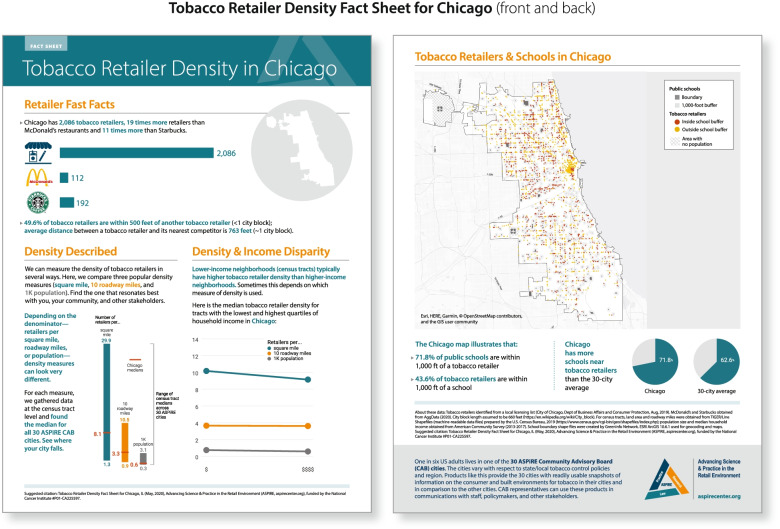
Example translational products from ASPiRE

Participants desired a number of different formats for receiving scientific evidence. Fact sheets summarizing the current scientific evidence for an issue were the primary request (See Fig. 3 as an example), although there was also strong interest in a number of other products including case studies describing how polices had been implemented in other locales, policy briefs summarizing the characteristics and potential impacts of specific tobacco control policies, and infographics about tobacco control and use. Of note, participants indicated almost no interest in full-length reports or scientific manuscripts. When asked with whom they would share ASPiRE products, participants indicated they would share this evidence with other audiences including city council members or other policy makers, community members, and staff within their own departments.

### Product Development

The D&I Core team developed a number of translational products showcasing the work of ASPiRE research teams and other emergent research in tobacco control. In selecting product formats, the team was responsive to CAB member requests for fact sheets and similar, more brief documents. In the first three years of the ASPiRE Center, the D&I core team published 10 presentations or posters, 90 city-specific fact sheets, and 4 brief reports.

In response to CAB preferences for evidence dissemination modes, all materials to date have been shared with the CAB through multiple channels, including by email to CAB members, via virtual CAB meetings, and by posting on the ASPiRE website. The ASPiRE website (aspirecenter.org) was launched in April 2020 as a communication and dissemination channel for sharing not only ASPiRE research findings, but also other retail policy resources, news, and updates from partner organizations. The website serves as an example of how our D&I Core has been responsive to CAB member feedback on several counts. First, ‘Internet’ was one of the top three channels identified in CAB member responses to the communication channel polling question. Second, the website houses a resource library that includes all types of materials requested by CAB members (e.g., fact sheets, case studies, and infographics) and was designed for a broad audience of individuals working in tobacco retail policy and research, including those identified by CAB members (e.g., tobacco control staff, policy makers, community members). And third, the website is an ideal platform for responsive sharing of timely, relevant, and tailored ‘News & Updates’ identified by ASPiRE team members, CAB members, and partners across the life of the ASPiRE project. 

While the website serves as both a channel for dissemination and a product, the D&I Core also developed a number of static products. As an example, the D&I Core collaborated with one of the ASPiRE’s research projects to develop three sets of city-specific products: 1)*Tobacco Retail Density Fact Sheets*, 2) *Tobacco Swamps Maps*, and 3) *Tobacco Sales Fact Sheets,* which provide detailed information about tobacco retail density and proximity to key locales, areas of heavy retailer concentration, and tobacco sales [29]. For the first and third products, the D&I Core informally solicited input and incorporated feedback from two CAB members to ensure the products were understandable and useful. In Fig. 3, we highlight the Retailer Density Fact Sheet, a product with high uptake and use by CAB members. The design of this brief, digestible, visually appealing, and user-friendly product demonstrates was based on the translational product design principles developed and codified by members of the study team (evidence-informed source, actionable information, modular design, understandable language, and accessible format). For the *Tobacco Sales Fact Sheets*, in keeping with active dissemination approaches of tailoring products for specific audiences, the D&I Core developed a one-page digest as a ‘cover’ to the 2 pages of detailed sales data. The CAB members who provided feedback shared their intention to use the detailed data sheets in their own internal planning and the one-page digest when speaking with policy makers. The D&I Core also worked with another ASPiRE research project to disseminate the Tobacco Swamps Dashboard, a city-specific, interactive dashboard which allows the user to project potential impacts of retailer policies for each locale (https://aspirecenter.org/tobacco-swamps/). All aforementioned products featured summary ‘fast facts’ about the data, data visualizations (e.g., charts, maps, and infographics), plain language interpretations of the findings, and ASPiRE branding.

### Product Dissemination and Engagement

On average, 61% (SD 7%) of CAB members opened the newsletter per month and 21% (SD 9%) clicked on a resource link within the newsletter. In our Fall 2020 CAB meeting, almost all (95%) CAB participants felt the newsletter was useful or very useful and was delivered at the right frequency, and 95% visited aspirecenter.org at least once in the first six months. Website analytics also suggest wider dissemination outside of CAB members. From April 2020 through July 2020 there were over 5,000 users and over 18,000 individual page views. Through the website, 46 additional (non-CAB) individuals signed up to receive communications from ASPiRE including ASPiRE eNews.

**Fig. 4:**
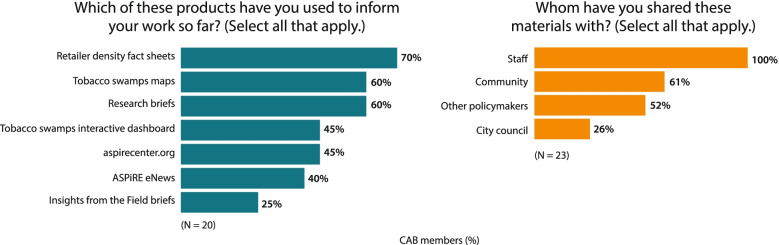
Product types used and stakeholders with whom CAB members shared, 2020

In addition to the website, a follow-up poll of CAB members in October of 2020 showed that 70% had used these products to inform their work. CAB members reported using a variety of additional ASPiRE products (Fig. 4, left). They also shared these products with several other stakeholders (Fig. 4, right), although these stakeholders differed slightly from their anticipated use reported in the first CAB meeting (Fig. 2).

Additionally, our dissemination efforts were increased through the Campaign for Tobacco-Free Kids (a CAB member organization) in a joint multi-city media campaign to focus attention on the proximity of tobacco retailers to schools and the high retail density in low-income neighborhoods. The reach of and audience engagement with the two-week campaign was substantial: over 3 million broadcast viewers, radio listeners, and on-line readers in more than two dozen of the nation’s largest media markets viewed or heard the information. After the campaign, we observed a four-fold increase in traffic to the ASPiRE website.

## Discussion

Similar to other areas of public health and social policy, the implementation of evidence-informed tobacco control policies remains the most effective and sustainable type of public health intervention to reduce tobacco use, tobacco-related disparities, and the public health burden of tobacco use. Policy evidence translation and dissemination are crucial for the development and promotion of evidence-informed policies yet remain understudied areas of research [11]. This study assessed dissemination and translational preferences as well as reported use of evidence among a group of local tobacco control leaders within the context of researcher-initiated dissemination efforts. Our research supports previous studies’ conclusions that policy audiences, including those who work in tobacco control policy, prefer to receive scientific evidence translated into formats that are shorter and accessible over formats more typical of academia like academic conference presentations or scientific manuscripts in scholarly journals [15, 19, 30]. While it was unsurprising that CAB members preferred online formats, this particular group had no interest in receiving information on social media. This could speak to a lagged adoption of social media or qualities about social media that make it an undesirable format among those who work in local tobacco control policy.

Successful tobacco control interventions have long relied upon appropriate and broad dissemination strategies [31, 32]. Our work extends the current literature by demonstrating how collaborating with practitioners to develop useful translational products can help to successfully disseminate evidence to intended audiences that serve important bridging roles to promote evidence-informed policymaking. By considering translational product design and D4D [25], we strived to make products tailored and succinct, as with our city-specific fact sheets for instance, which adhere to the preferences of our audience. In addition, eliciting feedback from those on the ground about their specific needs for evidence and product preferences given their local policy environment helped our team identify appropriate translational strategies, enhance the relevance of the evidence, and shorten the lag between scientific discovery an practice. Lastly, we also demonstrate how leveraging the D4DS framework can amplify dissemination. If materials are appropriate for a wide audience, it then becomes easy for collaborators or groups with similar interests to also disseminate these materials. In our experience, this resulted in exponential dissemination of materials that was far more than what our group could accomplish alone.

As funders and governments encourage attention to leveraging scientific evidence for better policy impact [33], there are several ways researchers can enhance the reach of their own work and replicate the process that we have described here. As we have demonstrated, community-research partnerships which integrate community perspectives and preferences early in the research process help improve the relevance and demand for evidence. Though the dissemination preferences of CAB members reported here may be relevant broadly to intersectoral partnerships for evidence-informed health policy dissemination and implementation, health policy researchers should also intentionally assess the contextual factors, needs, and preferences of their specific intended audience. We recommend a similar approach that leverages design principles [21]. We also recommend being as responsive as possible to preferences to expeditiously meet the demand from the intended audience for scientific evidence [34]. In our experience the benefit is threefold. First, it increases the chances that evidence will be used in the field, beyond academia. Second, it enhances the usability of the evidence and decreases the lag time we frequently observe from the generation of evidence and integration into policy and practice [35]. Third, responding to community preferences and requests creates a mutually beneficial, working partnership between scientists and communities that provides valuable insights for the scientific community into innovative policy work and trends that are happening on the ground and areas for future research.

For investigators who may be new to the field of dissemination for policymaking, there are several training opportunities to help become familiar with common dissemination frameworks, concepts, and approaches (like Designing for Dissemination) to help integrate their research into policy. Additionally, given the importance of community-based work to advancing health equity, it is important for researchers to know how to present and share information that will be used by—and shared by—community members [36–38]. We also recognize that it is often difficult for researchers, who are competing with other academic demands, to develop the skill set needed for product development. Our D&I core consists of a multidisciplinary team with varied expertise including tobacco control policy research, dissemination and implementation research, graphic design, website design, dashboard development, and journalism. We recommend that other investigators consider collaborating with individuals who hold similar expertise to facilitate dissemination efforts. Because funders, e.g., The National Institutes of Health, are increasingly supporting dissemination efforts, including or contracting individuals with additional skill sets that enhance translation and dissemination of evidence is now more feasible [39].

## Limitations

As a tenet of implementation science, context should be taken into consideration when planning dissemination and implementation efforts, and a one-size-fits-all approach is likely to only be minimally effective [40, 41].  The survey used in this study was developed by the ASPiRE Center as part of evaluation efforts and was not assessed for validity and reliability. Not all CAB members attended the meetings where the questionnaires were administered. There could be systematic differences in CAB members who were unable to participate, and tailoring communication and dissemination efforts to this population might promote engagement and evidence adoption. As such, the results reported here may not be generalizable to different communities. However, the process for gathering information to better understand preferences and developing products for dissemination is widely replicable.

## Conclusions

As global momentum for retail-based tobacco control policy interventions continues to grow, considering stakeholder preferences for how evidence is translated to those who can take policy action is increasingly important. Policy researchers in academic environments can enhance the value and impact of their work by promoting awareness, uptake, and integration of research findings in the generation of evidence-informed health policies. The approach taken here for surveying intended audiences and responding to feedback by tailoring dissemination products and efforts could serve as an exemplar for health policy researchers engaged in intersectoral partnerships for improved evidence translation and use in policy development.

## Data Availability

The datasets generated and analyzed during the current study are not publicly available due to the small size of the data set which may reveal particulars about specific individuals or locales.

## Abbreviations

ASPiRE: Advancing science and practice in the retail environment
CAB: Community advisory board
D&I: Dissemination and implementation
D4DS: Designing for dissemination and sustainability framework
